# p53-independent p21 induction by MELK inhibition

**DOI:** 10.18632/oncotarget.18488

**Published:** 2017-06-15

**Authors:** Tatsuo Matsuda, Taigo Kato, Kazuma Kiyotani, Yunus Emre Tarhan, Vassiliki Saloura, Suyoun Chung, Koji Ueda, Yusuke Nakamura, Jae-Hyun Park

**Affiliations:** ^1^ Department of Medicine, The University of Chicago, Chicago, IL, USA; ^2^ OncoTherapy Science Inc., Kawasaki, Japan; ^3^ Project for Realization of Personalized Cancer Medicine, Cancer Precision Medicine Center, Japanese Foundation for Cancer Research, Tokyo, Japan; ^4^ Department of Surgery, The University of Chicago, Chicago, IL, USA

**Keywords:** maternal embryonic leucine zipper kinase, molecular target, p21, p53, FoxO family

## Abstract

MELK play critical roles in human carcinogenesis through activation of cell proliferation, inhibition of apoptosis and maintenance of stemness. Therefore, MELK is a promising therapeutic target for a wide range of cancers. Although p21 is a well-known p53-downstream gene, we found that treatment with a potent MELK inhibitor, OTS167, could induce p21 protein expression in cancer cell lines harboring loss-of-function *TP53* mutations. We also confirmed that MELK knockdown by siRNA induced the p21 expression in p53-deficient cancer cell lines and caused the cell cycle arrest at G1 phase. Further analysis indicated that FOXO1 and FOXO3, two known transcriptional regulators of p21, were phosphorylated by MELK and thus be involved in the induction of p21 after MELK inhibition. Collectively, our herein findings suggest that MELK inhibition may be effective for human cancers even if *TP53* is mutated.

## INTRODUCTION

MELK (maternal embryonic leucine zipper kinase) is a cell-cycle dependent protein kinase and over expressed in various types of human cancer, but its expression in normal human organs is limited to testis and embryonic tissues [1–6]. We previously reported MELK as a promising therapeutic target and developed a potent MELK kinase inhibitor, OTS167, which showed strong antitumor effects in mice xenograft models of several cancer types [1, 4–8]. At present, therapeutic potential of OTS167 is evaluated in clinical trials (NCT01910545, NCT02795520, NCT02926690). MELK is shown to influence on several signaling pathways in cancer cell proliferation and survival, including the p53-p21 pathway [8, 9]. Indeed, Kig C et al. showed that siRNA-mediated MELK knockdown could activate the p53-p21 pathway and induced cell cycle arrest in glioblastoma cells [9].

We have characterized a part of the p53-signaling pathways in human cancer cells through identification of novel p53-target genes [10]. When the DNA damage occurs, the p53 is activated and causes cell cycle arrest at G1 phase by induction of a cyclin-dependent kinase inhibitor, p21 [10–12]. Although p53 is a major transcriptional factor of p21 and its mutation is associated with decreased p21 expression [13], the p53-independent induction of p21 has also been investigated by many groups [14]. For examples, BRCA1 was shown to activate p21 through both p53-dependent and -independent mechanisms [15], and Forkhead box O (FoxO) families could bind to a promoter region of the p21 gene (*CDKN1A*) and increase its transcriptional level [16–18]. In concordant with these previous reports, our previous study also showed a dose-dependent induction of p21 after treatment with OTS167, in the *TP53* wild-type cancer cells as well as *TP53*-mutated cancer cells [8].

In this study, we investigated molecular mechanism of the p53-independent induction of p21 by MELK inhibition. Our findings revealed that siRNA-mediated MELK knockdown increased protein levels of FOXO1 and FOXO3, which might increase p21 transcriptional level in a p53-independent manner. Since MELK could directly phosphorylate FOXO1 and FOXO3, our results further implied that effective restoration of these transcriptional factors by OTS167 treatment may be one of the important pathways to suppress proliferation of the p53-deficient cancer cells by MELK inhibition.

## RESULTS

### SiRNA-mediated MELK knockdown induces p21 in HCT116 p53 wild-type (p53 (+/+)) and null (p53 (-/-)) cell lines

**Figure 1:**
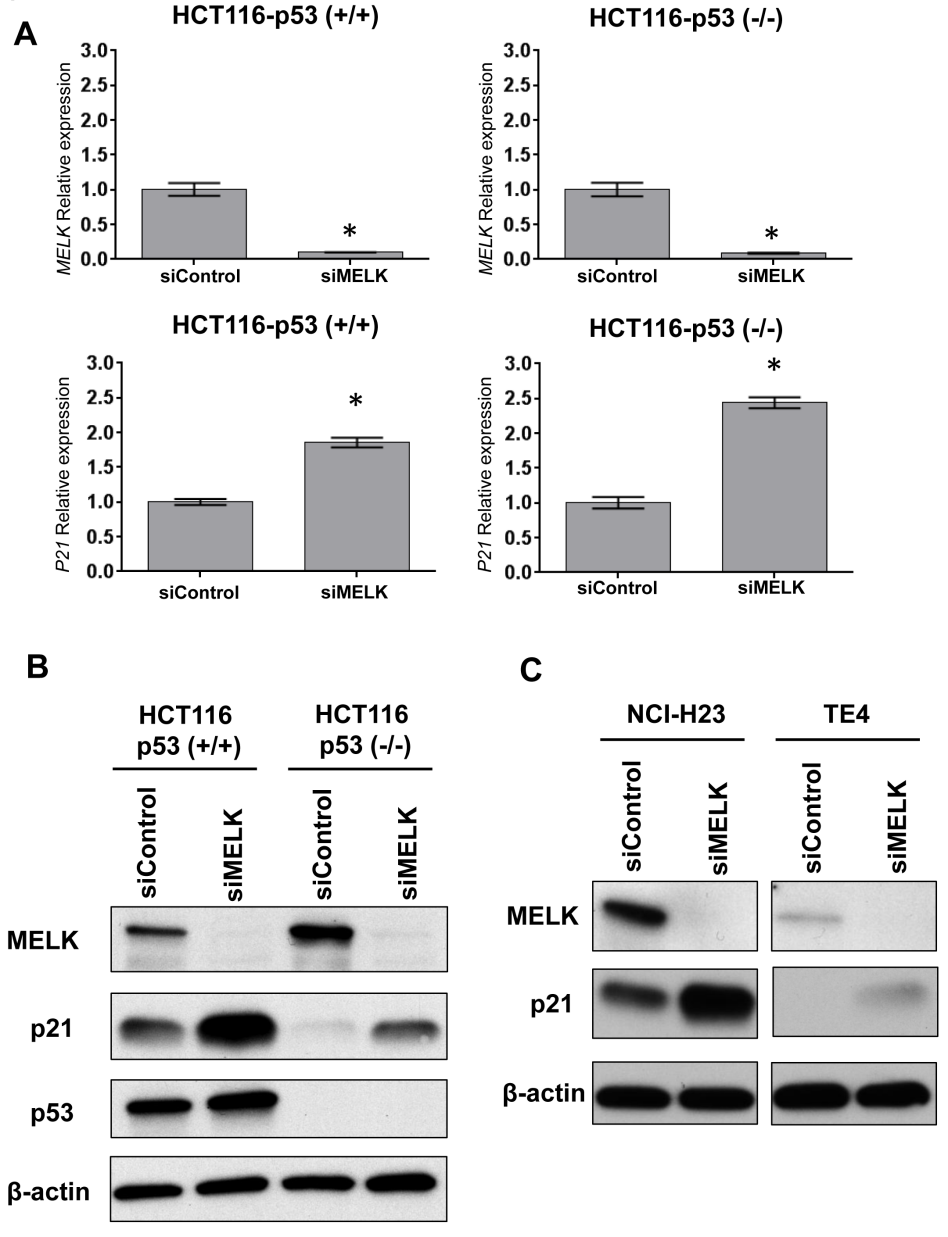
Knockdown effects of MELK in HCT116-p53 (+/+) and-p53 (-/-) cells **A**.Depletion of MELK and induction of p21 at transcriptional levels were observed in both cell lines by siRNA-mediated MELK knockdown. The asterisk indicates p < 0.01 compared with the corresponding value of the siControl group. **B**. Depletion of MELK and induction of p21 at protein levels were observed in both cell lines by siRNA-mediated MELK knockdown. **C**. TE4 and NCI-H23 cell lines harboring loss-of-function *TP53* mutations showed the increase of p21 protein after MELK knockdown.

To investigate whether MELK inhibition can induce p21 in a p53-independent manner, we transfected siRNA targeting MELK in HCT116 colon cancer cells with p53 wild-type, HCT116-p53(+/+), and its derivative p53-nulll cells HCT116-p53(-/-). Two days after transfection with siRNA, MELK was successfully depleted at transcriptional level (Figure 1A) and also protein level (Figure 1B) in both HCT116-p53(+/+) and -p53(-/-) cells. In this experiment, we also observed that significant induction of p21 at transcript level (Figure 1A) as well as protein level (Figure 1B) regardless of the p53 status although the induction level was higher in p53(+/+) cells than p53(-/-) cells. We further examined two additional cancer cell lines harboring loss-of-function *TP53* mutations, NCI-H23 (lung adenocarcinoma) and TE4 (esophageal squamous cell carcinoma), and observed increased protein level of p21 in these cell lines after MELK knockdown (Figure 1C). These results indicated that MELK could have an important role in transcriptional regulation of the p21 gene in a p53-independent pathway, in addition to transactivation of p21 through the p53 pathway.

### Stabilization of FOXO1 and FOXO3 after MELK knockdown

**Figure 2:**
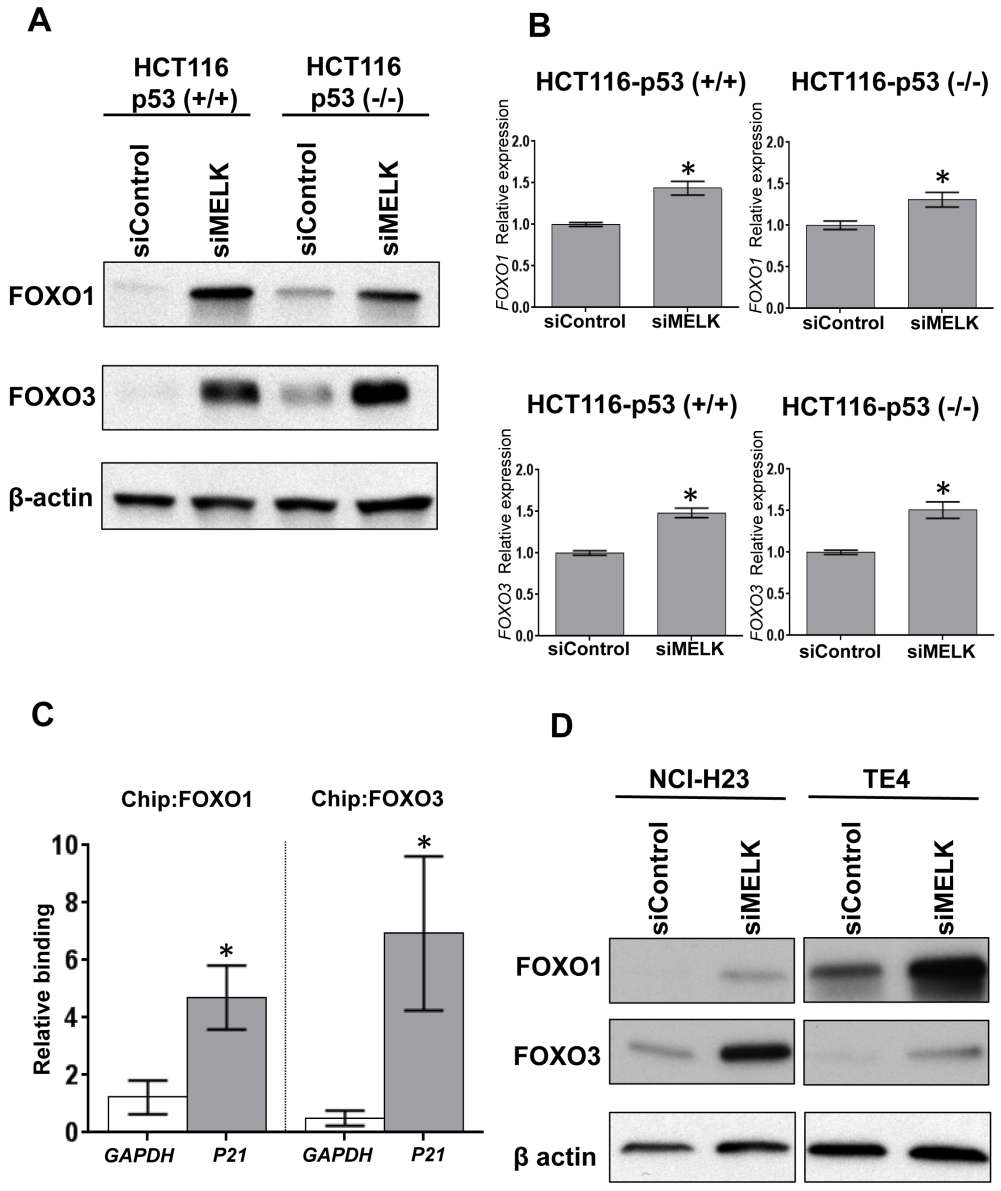
MELK knockdown increases FOXO1 and FOXO3 proteins **A**. FOXO1 and FOXO3 were increased at protein level in both HCT116-p53(+/+) and -p53(-/-) cells by siRNA-mediated MELK knockdown. **B**. FOXO1 and FOXO3 were also increased at transcriptional level in both HCT116-p53(+/+) and -p53(-/-) cells by siRNA-mediated MELK knockdown. The asterisk indicates p < 0.01 compared with the corresponding value of the siControl group. **C**. Using HCT116-p53(-/-) cells, chromatin immunoprecipitation (ChIP) and qPCR were performed to quantify FOXO1- or FOXO3-bound DNA complex on the promoter region of *p21* or *GAPDH* (negative control). The co-immunoprecipitated DNA of each antibody was normalized with that of normal IgG, and then its ratio of siMELK/siControl was calculated. The asterisk indicates *p* < 0.05 compared with the corresponding value of the *GAPDH*. **D**. NCI-H23 and TE4 harboring loss-of-function *TP53* mutations showed the increase of FOXO1 and FOXO3 proteins after MELK knockdown.

To investigate a possible transcriptional factor(s), which mediates the p53-independent induction of p21, we searched candidate transcriptional factors that might directly bind to the promoter region of the p21 gene using human reference genome GRCh37/hg19 assembly in the UCSC genome browser (http://genome.ucsc.edu). Through this analysis eight proteins (SRF, p53, FOXO1, FOXO3, FOXD1, FOXC1, FOXF2 and FOXJ2) were predicted as candidate transcriptional factors to bind to the promotor region in the p21 gene: among them, FOXO1 and FOXO3 were previously reported to bind to the p21 promoter region and induce the cell cycle arrest [16–18]. Hence, we firstly examined whether MELK knockdown affect the FOXO1 and FOXO3 protein levels in HCT116-p53(+/+) and -p53(-/-) cells. As shown in Figure 2A, MELK knockdown drastically increased both FOXO1 and FOXO3 protein levels in both of the HCT116 cells. Since their transcriptional levels were moderately increased by MELK knockdown (Figure 2B), we hypothesized that MELK knockdown might directly or indirectly influence on the stability of these proteins through post-transcriptional modifications. The p21 induction by MELK inhibition was abrogated by knockdown of either FOXO1 or FOXO3 in HCT116-p53(-/-) cell (Supplementary Figure S1). Particularly, knockdown of FOXO3 completely abrogated the p21 induction, indicating that FOXO3 is a much critical transcription factor to induce p21 when MELK was inhibited. In addition, chromatin immunoprecipitation assay revealed that MELK inhibition increased the amount of FOXO1 and FOXO3 proteins bound to the promoter region of the p21 gene in HCT116-p53(-/-) cells (Figure 2C). In addition to HCT116-p53(-/-) cells, we observed a similar effect (increased protein levels of FOXO1 and FOXO3) after MELK knockdown in another p53-deficient cancer cell lines, NCI-H23 and TE4 (Figure 2D). We also observed that treatment of MELK inhibitor (OTS167) increased FOXO1 and FOXO3 proteins, which led to induction of p21 in TE4 cell line (Supplementary Figure S2), but not clearly in NCI-H23 cell line. Since OTS167 inhibits multiple protein kinases in addition to MELK as reported previously [19], inhibition of other kinases by this inhibitor may affect the induction levels of p21.

### Cell cycle arrest at G1 phase by siRNA-mediated MELK knockdown

**Figure 3:**
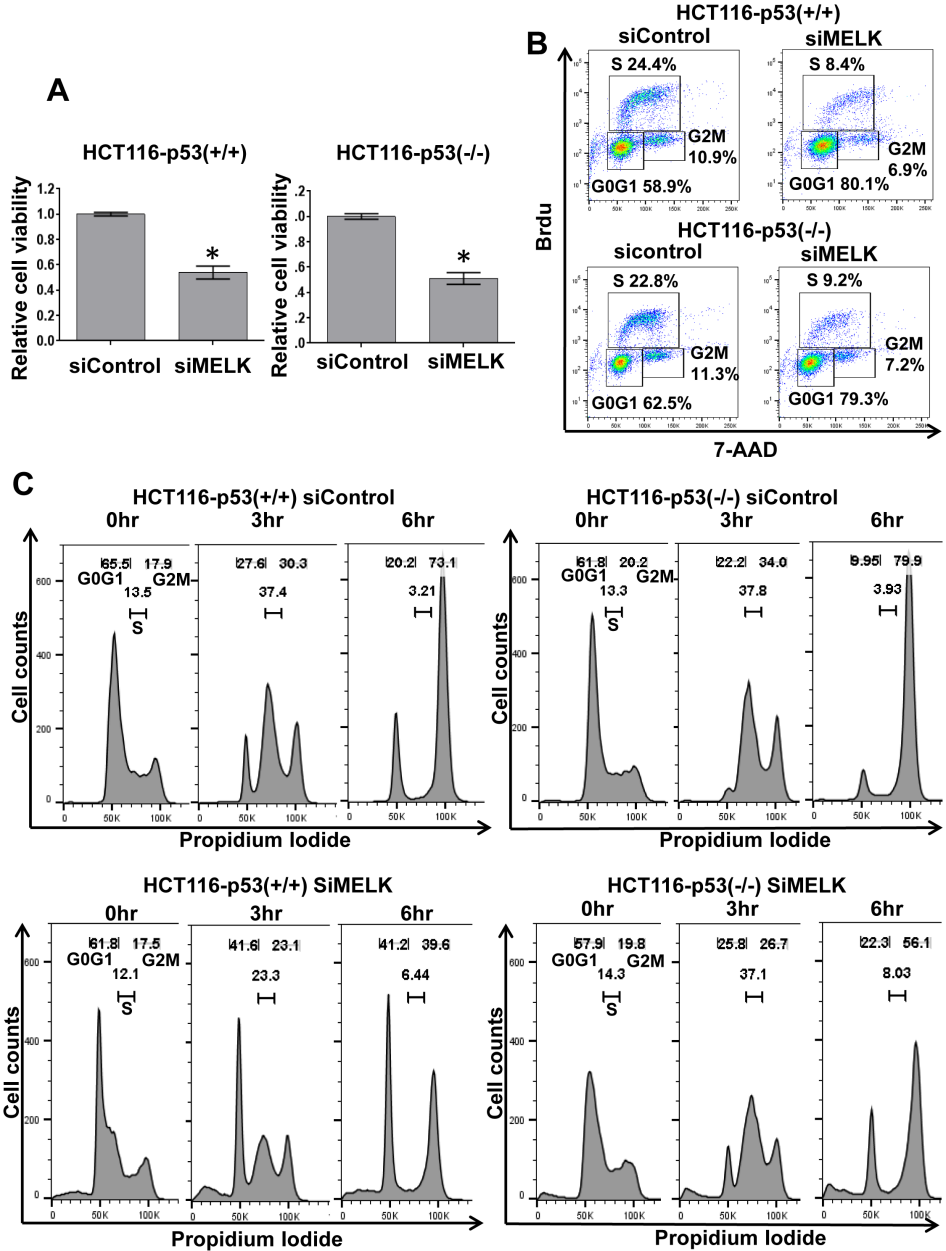
Cell cycle arrest at G1 phase by MELK knockdown **A**. Silencing of MELK by siRNA reduced the number of viable cells on both HCT116-p53(+/+) and -p53(-/-) cells. The asterisk indicates *p* < 0.01 compared with the corresponding value of the siControl group. **B**. Cell cycle analysis was performed in HCT116-p53(+/+) and -p53(-/-) cells treated with siControl and siMELK for 48 hours. Bromodeoxyuridin (Brdu) staining and 7-aminoactinomycin D (7AAD) were used to analyze the cell cycle. **C**. FACS analysis of HCT116-p53(+/+) and -p53(-/-) cells treated with siControl and siMELK for 48 hours. Cells were collected at 0, 3, and 6 hours after the release of cell cycle arrest caused by aphidicolin.

Because p21 was induced in both HCT116-p53(+/+) and -p53(-/-) cells, we examined the MELK knockdown effects on cell proliferation at day 6 by MTT assay and found significant decrease of viable cell number (p < 0.01) in the both HCT116 cells transfected with siMELK compared to those with siControl (Figure 3A). Induction of p21 is well known to cause the cell cycle arrest at G1 phase, thus we analyzed the proportion of cells at each cell-cycle phase after siRNA-mediated MELK knockdown in the both HCT116-p53(+/+) and -p53(-/-) cells (Figure 3B). At day 2 when MELK knockdown effect was observed, we performed bromodeoxyuridine (Brdu) incorporation assay and found the increase of the proportion of the cells at G0/G1 phase in both HCT116 cells (siControl vs siMELK; 58.9% vs 80.1% in p53(+/+) cells and 62.5% vs 79.3% in p53(-/-) cells, respectively). Concordantly, both HCT116 cells revealed the decrease in the proportion of the cells at S phase by MELK knockdown (siControl vs siMELK; 24.4% vs 8.4% in p53(+/+) cells and 22.8% vs 9.2% in p53(-/-) cells, respectively). To further validate these results, we performed cell-cycle analysis at 0, 3 and 6 hours after the release of cell-cycle arrest caused by aphidicolin (Figure 3C). Six hours after the cell cycle release, both HCT116-p53(+/+) and -p53(-/-) cells that were transfected with siMELK revealed higher proportions of the cells at G0/G1 phase compared to those with siControl (siControl vs siMELK; 20.2% vs 41.2% in p53(+/+) cells and 9.95% vs 22.3% in p53(-/-) cells, respectively). These results further confirmed that MELK knockdown induced the G1 arrest in the cells regardless of the p53 status.

### Direct phosphorylation of FOXO1 and FOXO3 by MELK

**Figure 4:**
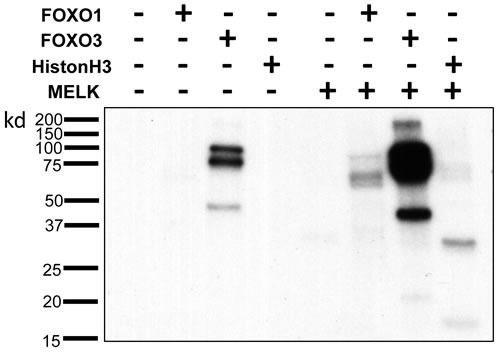
Direct FOXO1 and FOXO3 phosphorylation by MELK *In vitro* kinase assay of recombinant FOXO1 and FOXO3 proteins with MELK recombinant protein. Recombinant histone H3 protein was used as a positive control.

Since it was suggested that phosphorylation of FOXO1 and FOXO3 proteins could cause nuclear to cytoplasmic translocation and subsequent degradation of these proteins through the ubiquitin-proteasome pathway [16–18], we examined a possibility that MELK may directly phosphorylate FOXO1 and FOXO3 proteins. We performed *in vitro* kinase assay using recombinant proteins, and found that co-incubation of FOXO1 and FOXO3 with MELK increased the phosphorylation levels of these proteins (Figure 4 and Supplementary Figure S3) (Histone H3 protein was used as a positive control [19]). Because we observed a much stronger signal on FOXO3 protein than Histone H3 or FOXO1 protein, we assumed that MELK might phosphorylate multiple sites of the FOXO3 protein. FOXO3 showed phosphorylation signals without MELK recombinant protein (lane 3 of Figure 4) when the X-ray film was exposed for longer period, probably due to contamination of unknown protein kinase(s) during purification of recombinant FOXO3 protein. Since FOXO3 was intensively phosphorylated by MELK (Figure 4), we conducted mass spectrometry (MS) analysis for the *in vitro* phosphorylated FOXO3 protein and identified 23 MELK-dependent as well as 4 phosphorylation sites that were significantly-enhanced by MELK (Supplementary Table 1). Since the FOXO family members are known as direct substrates of AKT protein kinase, we further examined a possibility of AKT-mediated phosphorylation on FOXO1 and FOXO3 proteins. However, we could not see the any difference of total and phosphorylation levels (markers of AKT activity) of AKT protein in HCT116-p53(+/+) and -p53(-/-) cells after MELK knockdown (Supplementary Figure S4).

## DISCUSSION

MELK is overexpressed in various types of solid and hematological cancers, and has been reported to correlate with poor prognosis of cancer patients. Many groups including us have investigated critical roles of MELK in various processes of human carcinogenesis, such as proliferation, invasion, anti-apoptosis and stemness of cancer cells [1–9]. Since MELK was considered as an ideal therapeutic target for cancer treatments, we developed potent MELK inhibitors [7]. Among several signaling pathways affected by MELK inhibition, the p53-dependent induction of p21 was already reported. For example, Kig et al. reported that loss of MELK increased the p21 expression in glioblastoma cells and this p21 induction was mediated by the activated ATM (ataxia telangiectasia mutated)-Chk2-p53 pathway [9]. In addition to our group, Beke et al. developed another MELK inhibitor (MELK T1) that could induce a phosphorylation of p53 and then upregulate p21 [20]. We also showed that our MELK inhibitor (OTS167) activated p53 and p21 in the cancer cell line with wild-type p53, but unexpectedly we observed activation of p21 in the cancer cell lines even with p53 mutation [8], indicating a possibility of p53-independent p21 activation mechanism regulated by MELK in cancer cells.

In this study, we compared HCT116-p53(+/+) and -(-/-) cell lines and found that siRNA-mediated MELK knockdown could increase p21 proteins, regardless of the p53 status. This p21 induction was explainable for the cell cycle arrest at G1 phase in the p53 null HCT116 cell line under the MELK depletion condition by siMELK. We subsequently attempted to clarify the mechanism of p53-independent p21 induction and found two possible transcriptional factors, which might be responsible to activate the p21 transcription.

FoxO family members play tumor suppressive roles by activating multiple target genes [16]. Phosphorylation is known as one of important protein modifications on FoxO proteins since it affects the protein stability of FoxO through the ubiquitination-mediated proteasomal degradation pathway [16–18]. In chronic myelogenous leukemia, a tyrosine kinase inhibitor (imatinib) activated FOXO1 and FOXO3 by blocking the PI3K-AKT pathway, and in turn induced cell-cycle arrest and apoptosis [21]. Similarly, our results in this study indicated that FOXO1 and FOXO3 might be novel substrates of MELK and that restoration of FOXO1 and FOXO3 might be another mechanism to cause growth suppressive effects through MELK inhibition.

Our findings further implied that MELK is an ideal therapeutic target for treatment of cancer cells, regardless of the p53 status. Even in the p53-mutated cancer cells, we found that MELK inhibition induced p21 and suppressed the cell proliferation by causing G1 arrest. p21 is known to have a function to interact with PCNA and inhibit the DNA repair by modulating various DNA repair processes [22]. Indeed, p21 overexpression sensitizes ovarian cancer cell lines to cisplatin [23]. Therefore, MELK inhibitor in combination with the DNA-damaging therapies may effectively kill cancer cells, particularly be useful for those harboring *TP53* mutations.

In conclusion, MELK inhibition can induce p21 expression and cause G1 arrest in the p53-independent pathway, which is mediated probably by stabilization of FOXO1 and FOXO3. Our findings suggest that MELK inhibitor may be applicable to treatment of cancers regardless of the *TP53* status.

## MATERIALS AND METHODS

### Cell lines

HCT116 p53 wild-type (p53 (+/+)) and null (p53 (-/-)) isogenic colorectal cancer cell lines were kindly provided by Dr. Vogelstein (Johns Hopkins University, Baltimore, MD). The *TP53* genes were disrupted in HCT116-p53(-/-) cells by homologous recombination [24]. TE4 esophageal squamous cancer cell line was obtained from the Cell Resource Center for Biomedical Research Institute of Development, Aging and Cancer, Tohoku University (Sendai, Japan). NCI-H23 lung adenocarcinoma cell line was purchased from the American Type Culture Collection (ATCC) (Rockville, MD). Both HCT116-p53 (+/+) and -p53 (-/-) cells were cultured in DMEM media (Life Technologies, Grand Island, NY) with 10% FBS and 1% antibiotic-antimycotic solution (Sigma-Aldrich, St. Louis, MO). TE4 and NCI-H23 cells were cultured in RPMI media (Life Technologies) with 10% FBS and 1% antibiotic-antimycotic solution. All cells were maintained at 37 °C in humidified air with 5% CO_2_.

### Oligo siRNA and transfection

For knockdown experiments, cells were transfected with 200 pmol of oligo siRNA using Lipofectamine RNAiMAX (Invitrogen, Carlsbad, CA) according to manufacturer's instructions. The target sequences of each oligo siRNA were 5’-GACAUCCUAUCUAGCUGCA-3’ for MELK; 5’- GAGCGUGCCCUACUUCAAG-3’ for FOXO1 and 5’-CAACCUGUCACUGCAUAGU-3’ for FOXO3. For a control siRNA (siControl), SIC001 Mission siRNA Universal Negative Control was purchased from Sigma-Aldrich (St. Louis, MO).

### Western blot analysis and antibodies

Cells were lysed on ice with IP lysis buffer (Thermo Scientific, Waltham, MA) containing protease inhibitor cocktail set III (Millipore, Billerica, MA). Total proteins were separated by electrophoresis using Any kD precast polyacrylamide gel (Bio-Rad, Hercules, CA), and transferred onto PVDF membrane. After blocking with 5% skim milk (Thermo Scientific) in TBST buffer, membranes were incubated with the first antibody, respectively: anti-MELK monoclonal antibody (in-house, previously described [8]), anti-β-actin antibody, anti-p21 antibody, anti-FOXO1 antibody, anti-FOXO3 antibody, anti-pan-AKT antibody, anti-phospho-AKT (Thr308) antibody, anti-phospho-AKT (Ser473) antibody (Cell Signaling, Danvers, MA), and anti-p53 antibody (Sigma-Aldrich). β-actin was used as a loading control.

### Cell viability assay

Cancer cells were seeded into 24-well flat-bottom plates at 5× 10^4^ cells per well, and mixed with oligo siMELK using Lipofectamine RNAiMAX (Invitrogen). Three days later, cancer cells were transfected again with oligo siMELK to maintain MELK knockdown effects. Cells were cultured at 37°C under 5% CO_2_ for 6 days from first transfection. The Cell counting kit-8 (Dojindo Molecular Technologies, Inc., Kumamoto, Japan) was used for MTT reaction and examined the cell viability. After reaction for 1 to 3 hr, 100 μL of supernatant was transferred into a 96-well plate and read at 450 nm using the iMark microplate reader (Bio-Rad). All of these experiments were done in triplicate.

### Real-time RT-PCR

Total RNA was extracted from cancer cells using RNeasy Mini Kit (Qiagen, Valencia, CA), then reversely transcribed using SuperScript III First-Strand Synthesis System (Invitrogen) following the manufacturer's instructions. Aliquots of the reverse transcription product were quantified by real-time RT-PCR. The real-time RT-PCR was performed using primers listed below in the ViiA 7 system (Life Technologies). The PCR primer sequences were 5’-GCTGCAAGGTATAATTGATGGA-3’ and 5’-CAGTAACATAATGACAGATGGGC-3’ for *MELK*; 5’- GGAAGACCATGTGGACCTGT-3’ and 5’-GGCGTTTGGAGTGGTAGAAA-3’ for *p21*; 5’-CGACCACTTTGTCAAGCTCA-3’ and 5’-GGTTGAGCACAGGGTACTTTATT-3’ for *GAPDH*; 5’-TCGTCATAATCTGTCCCTACACA-3’ and 5’-CGGCTTCGGCTCTTAGCAAA-3’ for *FOXO1*; 5’-CGGACAAACGGCTCACTCT-3’ and 5’-GGACCCGCATGAATCGACTAT-3’ for *FOXO3.* Finally, expression level of each gene was normalized with that of *GAPDH*.

### Cell cycle analysis

For the Brdu incorporation assay, cells were transfected with siControl or siMELK for 48 hours, and then cell cycle was analyzed by BD Pharmingen FITC Brdu Flow kit (Becton Dickinson, San Jose, CA) according to manufacturer's instructions. Fluorescence signal was quantified by flow cytometry (FACS LSRII; Becton Dickinson) using Flow Jo software (Treestar, Ashland, OR). To synchronize cell cycle at the G0/G1 phase, cells were exposed to aphidicolin (5 μg/mL) for 24 hr and then the cell cycle was released by PBS washing. Finally, cells were collected by trypsinization after the culture of 0, 3 and 6 hours, fixed in 70% cold ethanol, and followed by treatment with RNase and propidium iodide in PBS for the FACS analysis.

### *In vitro* kinase assay

MELK recombinant protein was kindly obtained from OncoTherapy Science Inc. As a control for the *in vitro* kinase assay, Histone H3 recombinant protein (EMD Millipore) was used as a positive control. In each reaction, 0.15 μM of FOXO1 (EMD Millipore), FOXO3 (Abnova, Taipei, Taiwan), or Histone H3 recombinant protein was mixed with 0.15 μM of MELK recombinant protein in 50 μl of kinase buffer and incubated for 2 hours at 30 °C. The kinase buffer contained 50 mM Tris-HCl, 10 mM NaCl, 10 mM MgCl_2_, 10 mM NaF, 1 mM Na_3_VO_4_, 1 mM DTT, 0.1 mM EDTA with 50 μM cold-ATP and 10 mCi of [γ-^32^P]ATP (GE Healthcare). The reaction was terminated by addition of SDS sample buffer and boiled for 5 min. Finally, the reacted samples were electrophoresed on Any kD precast polyacrylamide gel (Bio-Rad), transferred onto the PVDF membrane, and then autoradiographed with X-ray films.

### Identification of FOXO3 phosphorylation sites

In the same manner with *in vitro* kinase assay, 0.15uM recombinant FOXO3 protein (Abnova) was mixed with 0.15 μM of MELK recombinant protein in 50 μl of kinase buffer and incubated for 2 hours at 30 °C. The kinase buffer contained 50 mM Tris-HCl, 10 mM NaCl, 10 mM MgCl_2_, 10 mM NaF, 1 mM Na_3_VO_4_, 1 mM DTT, 0.1 mM EDTA with 500 μM cold-ATP. The reaction was terminated by addition of SDS sample buffer and boiled for 5 min. The reactant was separated on SDS-PAGE and stained by CBB-staining. The excised FOXO3 bands were resolved in 10 mM tris(2-carboxyethyl)phosphine (Sigma-Aldrich) with 50 mM ammonium bicarbonate (Sigma-Aldrich) for 30 min at 37°C and alkylated in 50 mM iodoacetamide (Sigma-Aldrich) with 50 mM ammonium bicarbonate for 45 min in the dark at 25°C. Trypsin/Lys-C (Promega) solution was added and incubated at 37°C for 12 h. The resulting peptides were extracted from gel fragments and analyzed with Orbitrap Fusion Lumos mass spectrometer (Thermo Scientific) combined with UltiMate 3000 RSLC nano-flow HPLC system (Thermo Scientific) with HCD or EThcD MS/MS mode. The MS/MS spectra were searched against Homo sapiens protein sequence database in SwissProt using Mascot or Sequest search engine in Proteome Discoverer 2.1 software (Thermo Scientific), in which peptide identification filters were set at “false discovery rate < 1%” and “Mascot expectation value < 0.05 or Sequest XCorr > 2.0”.

### Chromatin immunoprecipitation assay

Chromatin immunoprecipitation (ChIP) assay was performed using a ChIP Assay kit (Millipore) according to the manufacturer's protocol. Briefly, FOXO1/FOXO3 and fragmented chromatin complexes were immunoprecipitated with 10 μg each of anti-FOXO1 (Abcam), anti-FOXO3 (Thermo Scientific), or normal rabbit IgG (Santa Cruz Biothechnology, Santa Cruz, CA) antibody, 48 hours after MELK knockdown. Co-immunoprecipitated DNA fragments were quantified using primers listed below in the ViiA 7 system (Life Technologies). The ChIP qPCR primer sequences targeting gene promoter regions were 5’-TGTGAAGCTCAGTACCACAAAAA-3’ and 5’-AGGGCTGGTTGTCAAATGTC-3’ for *p21*; 5’-TACTAGCGGTTTTACGGGCG-3’ and 5’-TCGAACAGGAGGAGCAGAGAGCGA3’ for *GAPDH* (negative control).

### Statistical analysis

Data were presented as mean ± one standard deviation. Differences between two groups were calculated for significance using student's *t* test, and *p* < 0.05 was considered as statistically significant.

## SUPPLEMENTARY MATERIALS FIGURES AND TABLES




