# Supplementary material for: p53-independent p21 induction by MELK inhibition
Source: Oncotarget. 2017 Jun 15;8(35):57938–47. doi: 10.18632/oncotarget.18488 (PMC5601624; doi:10.18632/oncotarget.18488)
Supplement: Supplementary file 1 [file oncotarget-08-57938-s001.pdf]

p53-independent p21 induction by MELK inhibition

Supplementary Material

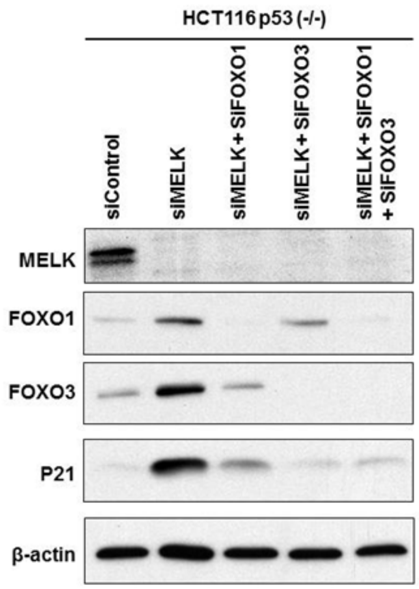

Supplementary Figure 1: p21 induction by MELK inhibition was abrogated by knockdown of FOXO1 and/or FOXO3. Combination of FOXO1 and/or FOXO3 knockdown with MELK knockdown abrogated the p21 induction by MELK inhibition alone.

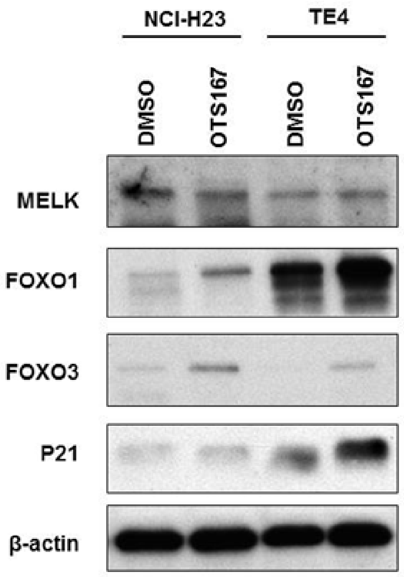

Supplementary Figure 2: Induction of FOXO1, FOXO3, and p21 by OTS167 treatment. NCI-H23 and TE4 cells were treated with OTS167 at the concentration of their IC_{50} values (9.2 nM for NCI-H23, 25.7 nM for TE4) for 24 hrs. Treatment with OTS167 increased FOXO1 and FOXO3 protein levels in both NCI-H23 and TE4 cells. The induction of p21 was clearly detected in TE4 cells, but not in NCI-H23 cells.

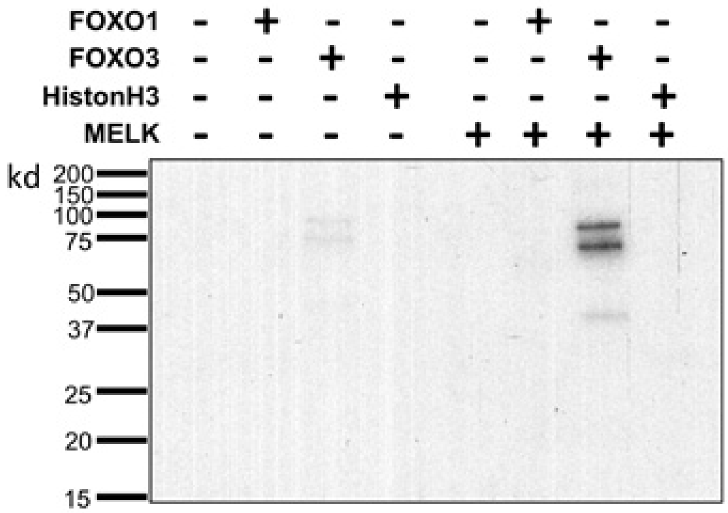

Supplementary Figure 3: ***In vitro*** **kinase assay results.** Short time exposure of X-ray film with a PVDF membrane containing recombinant proteins, which is corresponding to Figure 4. *In vitro* kinase assay of recombinant FOXO1 and FOXO3 proteins with MELK recombinant protein. Recombinant histone H3 protein was used as a positive control. FOXO3 protein was much highly phosphorylated than FOXO1 or histone H3 by MELK protein.

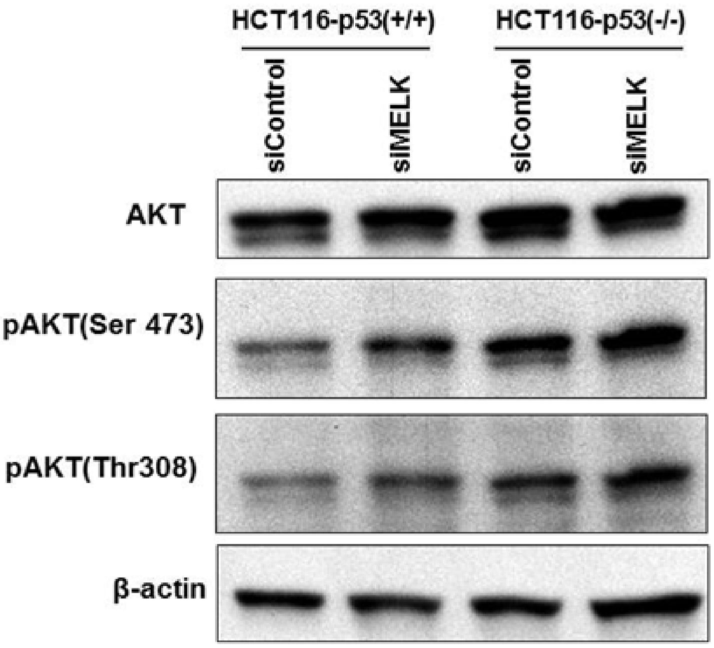

Supplementary Figure 4: No effect on AKT protein and phosphorylation levels by MELK knockdown. Total protein and phosphorylation levels of AKT were not affected by MELK knockdown in in either HCT116-p53(+/+) or -p53(-/-). Western blot images of β-actin were identical with those of Figure 2A because they were obtained from the same PVDF membrane.

For Supplementary Table see in Supplementary Files
